# A Case-Control Study of the Protective Effect of Alcohol, Coffee, and Cigarette Consumption on Parkinson Disease Risk: Time-Since-Cessation Modifies the Effect of Tobacco Smoking

**DOI:** 10.1371/journal.pone.0095297

**Published:** 2014-04-30

**Authors:** Marianne van der Mark, Peter C. G. Nijssen, Jelle Vlaanderen, Anke Huss, Wim M. Mulleners, Antonetta M. G. Sas, Teus van Laar, Hans Kromhout, Roel Vermeulen

**Affiliations:** 1 Division of Environmental Epidemiology, Institute for Risk Assessment Sciences, Utrecht University, Utrecht, the Netherlands; 2 Department of Neurology, St Elisabeth Hospital, Tilburg, the Netherlands; 3 Department of Neurology, TweeSteden Hospital, Tilburg, the Netherlands; 4 Department of Neurology, Canisius-Wilhelmina Hospital, Nijmegen, the Netherlands; 5 Department of Neurology, Vlietland Hospital, Schiedam, the Netherlands; 6 Department of Neurology, University Medical Center, Groningen, the Netherlands; 7 Julius Centre for Health Sciences and Primary Care, University Medical Center, Utrecht, the Netherlands; Centre Hospitalier Universitaire Vaudois (CHUV), Switzerland

## Abstract

The aim of this study was to investigate the possible reduced risk of Parkinson Disease (PD) due to coffee, alcohol, and/or cigarette consumption. In addition, we explored the potential effect modification by intensity, duration and time-since-cessation of smoking on the association between cumulative pack-years of cigarette smoking (total smoking) and PD risk. Data of a hospital based case-control study was used including 444 PD patients, diagnosed between 2006 and 2011, and 876 matched controls from 5 hospitals in the Netherlands. A novel modeling method was applied to derive unbiased estimates of the potential modifying effects of smoking intensity, duration, and time-since-cessation by conditioning on total exposure. We observed no reduced risk of PD by alcohol consumption and only a weak inverse association between coffee consumption and PD risk. However, a strong inverse association of total smoking with PD risk was observed (OR = 0.27 (95%CI: 0.18–0.42) for never smokers versus highest quartile of tobacco use). The observed protective effect of total smoking was significantly modified by time-since-cessation with a diminishing protective effect after cessation of smoking. No effect modification by intensity or duration of smoking was observed indicating that both intensity and duration have an equal contribution to the reduced PD risk. Understanding the dynamics of the protective effect of smoking on PD risk aids in understanding PD etiology and may contribute to strategies for prevention and treatment.

## Introduction

Coffee, alcohol and cigarette consumption are potential protective factors for the development of Parkinson disease (PD) [1]. The evidence for an association between cigarette smoking and PD risk is particularly strong with studies consistently showing an exposure dependent reduction in risk with total lifetime exposure to cigarette smoke, the product of (average daily) smoking intensity and duration of smoking [2]–[4]. However, it has been suggested that duration of smoking and time-since-cessation may be more relevant to PD risk than smoking intensity [5], [6]. Insight into the relative importance of duration, intensity, and time-since-cessation of cigarette smoking on PD risk may offer important clues to PD etiology and to strategies for prevention and treatment [5].

Due to their interrelatedness, modeling independent effects of duration, intensity and time-since-cessation of smoking on disease risk is complex. For example, when modeling the duration of smoking the parameter estimate represents the risk per year of smoking for a fixed intensity. As such the risks modeled at two different durations do not only reflect the difference in duration, but also the difference in total lifetime smoking. To circumvent this problem Lubin et al. described a model in which the modifying effects of intensity or duration can be investigated by conditioning the model on total exposure, allowing a comparison of the risk of low intensity exposures at long duration with risk of high intensity exposures at short duration [7]. Recently, this method was extended by including a term for time-since-cessation of smoking [8].

In a recently conducted hospital based case-control study we assessed the potential protective effect of coffee, alcohol and cigarette consumption on PD risk. The protective effect of cigarette smoking on PD risk was further explored by investigating the independent contribution of duration, intensity and time-since-cessation of smoking.

## Materials and Methods

### Ethics statement

The study was approved by the Medical Ethics Committee of St Elisabeth Hospital Tilburg, the Netherlands. All participants gave written informed consent.

### Cases and controls

Cases and controls were recruited between April 2010 and June 2012 from 5 hospitals in 4 different areas in the Netherlands. Eligible study subjects were identified using DBC codes, which is the standardized accounting system for hospital care based on diagnostic groups in The Netherlands [9]. In each hospital one neurologist reviewed the medical files of all subjects identified with DBC codes 0501 (PD) or 0502 (other extrapiramidal disorders) between January 2006 to December 2011. Subjects with an initial diagnosis before January 2006 or initially diagnosed elsewhere and referred to one of the participating centers for follow-up care or second opinion were excluded. Diagnoses included were: Parkinson disease, Progressive supranuclear palsy, Multiple system atrophy, Vascular Parkinsonism, Corticobasal degeneration and Dementia with Lewy bodies. For each included case, two matched controls were selected from individuals who attended the same departments of neurology within the same specified time-frame with DBC codes 0801 (median nerve neuropathy; ICD-10 G56.0 and G56.1), 0802 (ulnar nerve neuropathy; ICD-10 G56.2), 1203 (thoracic and lumbar disc disease; ICD 10 G55.1, G54.3 and G54.4) or 1204 (sciatica; ICD-10 M54.3 and M54.4). Controls were incidence density matched to the cases on hospital, visiting date (within 3 years of the cases diagnose year), sex and age (interquartile range age difference: 6–33 days, max: 512 days). As is standard in incidence density matching a control could serve as a control for more than one case [10].

### Telephone interview

Cases and controls were contacted via an invitation letter containing study information and a reply form for giving informed consent or to decline study participation. Non-responders were sent a reminder after one month, and one phone call attempt was performed after another month. Cases and controls were informed that the study objective was to study risk factors for neurological disorders, without specification. In a standardized computer-assisted telephone interview, participants were interviewed by one of three trained interviewers. The questionnaire contained a complete residential and occupational history, questions about selected dietary items, anthropometric measures and a medical history. Detailed questions about smoking behavior were asked to those reporting to have smoked more than 100 cigarettes during their lifetime. The questions about smoking covered start and final year of cigarette smoking and estimated amount of cigarettes smoked in 10 year periods. In addition, information on cigar or pipe smoking was obtained. Alcohol and coffee consumption was ascertained as glasses or cups per week at current age and at the age of 20, 40 and 60. For alcohol consumption information on binge drinking was obtained (i.e. 5 or more alcohol consumptions per occasion at least once a month). A typical telephone interview lasted 30 to 45 minutes.

### Exposure data

Smoking duration was defined as the number of years from start of smoking until year of diagnosis or year of smoking cessation, corrected for years not smoked in between. For former smokers time-since-cessation was defined as year of diagnosis minus year of smoking cessation. Intensity was defined as the average amount of cigarettes per day in the period of smoking. Total smoking was expressed as pack-years and calculated by dividing the intensity by 20 multiplied by the duration. Duration and intensity of alcohol and coffee consumption were estimated with the information on current consumption and consumption at the ages of 20, 40, and 60 (if relevant). Cumulative alcohol and coffee consumption was calculated as consumption-years by multiplying the average amount of consumptions per day with the estimated number of years of consumption.

### Statistical analysis

Due to the limited number of subjects with another Parkinsonism than PD (n = 40) we restricted our analyses to confirmed cases of PD (n = 444).

We estimated main effects (odds ratios (OR) and 95% confidence intervals (CI)) for total smoking, smoking intensity (cigarettes per day), time since cessation, total alcohol consumption, alcohol intensity (drinks per day), binge drinking (>4 drinks per day), total coffee consumption, and coffee intensity (drinks per day) with a conditional logistic regression model. Never users constituted the reference categories while users were divided based on the quartiles of the exposure distribution among the controls. The number of never users for coffee consumption was low (n = 38) and therefore the first quartile of coffee consumption (including the never users) was used as the reference category.

To investigate the potential modification of the relationship between total smoking and PD by intensity, duration and/or time-since-cessation of smoking, we applied an inverse excess OR model for total smoking, including modifying functions for smoking intensity or duration and time-since-cessation. The models described in this manuscript fall within a general framework for flexible modeling of the effects of intensity, duration, and time since exposure [8].

The model can be described as:
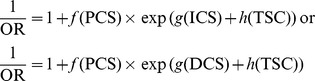



Where f(PCS) is a function of total pack-years of cigarette smoking modified by a function of smoking intensity g(ICS) or smoking duration g(DCS) and a function of time-since-cessation h(TSC). Because PCS is defined as ICS*DCS, models 1 and 2 yield a similar inference. f(PCS), g(ICS), g(DCS) and h(TSC) were included as linear function or as three-knot restricted cubic spline (knots located at the 20th, 50th, and 80th percentile). To compare the relative importance of duration, intensity, and time-since-cessation of cigarette smoking, model fit was evaluated based on the Akaike information criterion (AIC).

These models were fitted using the NLMIXED procedure in SAS v 9.2 (SAS Institute Inc., Cary, NC). Bootstrapped 95% confidence intervals were estimated via 100 bootstrap replications of the original data and taking the 2.5th and 97.5th percentiles of the resulting distribution. For presentation purposes the 1/OR were back-transformed to the OR.

In ancillary analyses we assessed the variation of ORs jointly by total smoking and time-since-cessation, by conducting unconditional logistic regression adjusted for the matching variables age, sex and center. In these analyses we categorized total smoking and time-since-cessation into quintiles and estimated ORs for crossed categories. All ORs were estimated relative to never smokers. We conducted unconditional rather than conditional logistic regression as case-control sets were broken due to the cross-categorization.

## Results

1,330 subjects with an initial diagnosis of Parkinsonism between 2006 and 2011 were identified. 1,220 (92%) of those were still alive at time of recruitment of which 1,001 had a diagnosis of PD. Ten current addresses were unknown, 530 persons declined participation and 192 did not reply. The number of successfully enrolled cases was 488 of which 448 were diagnosed with PD. Participation rate among PD cases was 45%. Among controls the participation rate was 35%. For 12 PD cases only 1 suitable control was found. For 4 PD cases no suitable controls were found and these were consequently excluded from the analysis. Table 1 shows the demographic characteristics of the 444 PD cases and 876 controls (of which 779 were unique) included in the present analyses.

**Table 1:** General Characteristics of Parkinson Disease Cases and Hospital Controls.

	Cases	Controls
	(n = 444)	(n = 876)
Men, No (%)	281 (63.3)	557 (63.6)
Age at interview, median (range)	68 (34–91)	68 (34–90)
Age at diagnosis, median (range)	67 (34–90)	-
Higher education^a^, No. (%)	268 (60.5)	477 (54.5)
Ever smoking cigarettes, No. (%)	237 (53.4)	633 (72.3)
Ever regular coffee consumption^b^, No. (%)	427 (96.2)	855 (97.7)
Ever regular alcohol consumption, No. (%)	340 (76.6)	679 (77.5)

a Information on education was missing for one case.

b Information on coffee consumption was missing for one control.

Cigarette smoking was inversely related with PD. 53% of the cases were ever smoker as compared to 72% of the controls (χ^2^ = 45.7, *P*<0.0001). When including cigar and pipe smoking these numbers changed only slightly to 57% and 75%, respectively. At the moment of interview 4% of the cases and 15% of the controls were still smoking cigarettes.

### Conditional logistic regression

We observed an inverse association between total pack-years of smoking, longer smoking duration, and shorter time-since-cessation and PD risk (table 2). For average smoking intensity, we observed reduced ORs among the exposed, but no trend was observed when limiting the analyses to ever smokers (*P* = 0.20).

**Table 2:** Parkinson Disease and Cigarette Smoking: Conditional Logistic Regression Analysis on Data of Patients and Hospital Controls.

	Cases	Controls	Crude	Adjusted^a^
	No. (%)	No. (%)	OR (95% CI)	OR (95% CI)
Total smoking (pack-years)				
Never smokers	207 (46.6)	243 (27.7)	1	1
>0–7.8	86 (19.4)	161 (18.4)	0.58 (0.42–0.82)	0.58 (0.42–0.82)
>7.8–17.5	67 (15.1)	155 (17.7)	0.45 (0.32–0.65)	0.46 (0.32–0.66)
>17.5–29.4	45 (10.1)	160 (18.3)	0.28 (0.18–0.42)	0.28 (0.18–0.42)
>29.4–103	39 (8.8)	157 (17.9)	0.26 (0.17–0.40)	0.27 (0.18–0.42)
*P* value for trend^b^			<0.0001/0.0004	<0.0001/0.0006
Smoking intensity (cigarettes/day)				
Never smokers	207 (46.6)	243 (27.7)	1	1
>0–7.0	77 (17.3)	158 (18.0)	0.53 (0.37–0.75)	0.53 (0.38–0.76)
>7.0–12.7	54 (12.2)	159 (18.2)	0.37 (0.25–0.53)	0.37 (0.25–0.54)
>12.7–19.2	50 (11.3)	158 (18.0)	0.33 (0.23–0.49)	0.34 (0.23–0.51)
>19.2–60.0	56 (12.6)	158 (18.0)	0.38 (0.26–0.54)	0.39 (0.27–0.57)
*P* value for trend^b^			<0.0001/0.16	<0.0001/0.20
Smoking duration (years)				
Never smokers	207 (46.6)	243 (27.7)	1	1
>0–18	98 (22.1)	165 (18.8)	0.66 (0.48–0.91)	0.66 (0.48–0.91)
>18–28	56 (12.6)	152 (17.4)	0.37 (0.25–0.54)	0.36 (0.24–0.53)
>28–41	48 (10.8)	166 (18.9)	0.29 (0.19–0.43)	0.29 (0.20–0.44)
>41–66	35 (7.9)	150 (17.1)	0.24 (0.15–0.37)	0.25 (0.16–0.39)
*P* value for trend^b^			<0.0001/<0.0001	<0.0001/<0.0001
Time-since-cessation (years)				
Never smokers	207 (46.6)	243 (27.7)	1	1
>31–53	93 (20.9)	158 (18.0)	0.67 (0.47–0.95)	0.65 (0.46–0.93)
>19–31	68 (15.3)	147 (16.8)	0.52 (0.36–0.76)	0.53 (0.36–0.77)
>0–19	54 (12.2)	166 (18.9)	0.35 (0.24–0.51)	0.36 (0.25–0.52)
0	22 (5.0)	162 (18.5)	0.15 (0.09–0.24)	0.15 (0.09–0.25)
*P* value for trend^b^			n.a./<0.0001	n.a./<0.0001

Abbreviations: OR = odds ratio, CI = confidence interval.

a The adjusted model includes coffee consumption (in quartiles).

b The first *P* value for trend was based on analyses with the exposure as a continuous variable including the persons from the reference category. The second p value for trend was based on analysis whereby the persons from the reference category are excluded.

Crude ORs for high total and average daily coffee consumption point towards a lower risk of PD (table 3), but were not statistically significant after adjustment for smoking. Analyses conducted among never smokers showed a similar non-significant trend for coffee consumption (ORs for the three highest quartiles of total coffee consumption among never smokers: OR = 1.11 (95% CI: 0.55, 2.26), OR = 0.97 (95% CI: 0.43, 2.16) and OR = 0.75 (95% CI: 0.34, 1.66)).

**Table 3:** Parkinson Disease and Coffee and Alcohol Consumption: Conditional Logistic Regression Analysis on Data of Patients and Hospital Controls.

	Cases	Controls^a^	Crude	Adjusted^b^
	No. (%)	No. (%)	OR (95% CI)	OR (95% CI)
Coffee consumption (Consumption-years)				
0–97	128 (28.8)	220 (25.1)	1	1
>97–156	146 (32.9)	221 (25.3)	1.13 (0.84–1.53)	1.30 (0.95–1.77)
>156–214	90 (20.3)	216 (24.7)	0.70 (0.50–0.97)	0.79 (0.56–1.12)
>214–720	80 (18.0)	218 (24.9)	0.61 (0.43–0.87)	0.83 (0.57–1.21)
*P* value for trend^c^			0.0016/0.0060	0.33/0.28
Coffee intensity (consumptions/day)				
0–2.1	129 (29.1)	223 (25.5)	1	1
>2.1–3.4	134 (30.2)	215 (24.6)	1.07 (0.79–1.45)	1.16 (0.85–1.59)
>3.4–4.7	103 (23.2)	218 (24.9)	0.79 (0.57–1.10)	0.92 (0.65–1.30)
>4.7–17.1	78 (17.6)	219 (25.0)	0.60 (0.42–0.85)	0.80 (0.55–1.16)
*P* value for trend^c^			0.0002/0.0020	0.10/0.12
Alcohol consumption (consumption-years)				
Never drinkers	104 (23.4)	197 (22.5)	1	1
>0–21	93 (20.9)	170 (19.4)	1.05 (0.74–1.49)	1.10 (0.75–1.60)
>21–48	99 (22.3)	172 (19.6)	1.10 (0.77–1.56)	1.44 (0.99–2.11)
>48–	80 (18.0)	169 (19.3)	0.87 (0.60–1.27)	1.27 (0.84–1.91)
>87–457	68 (15.3)	168 (19.2)	0.75 (0.50–1.11)	1.28 (0.82–1.98)
*P* value for trend^c^			0.14/0.18	0.38/0.73
Alcohol intensity (Consumptions/day)				
Never drinkers	104 (23.4)	197 (22.5)	1	1
>0–0.6	98 (22.1)	169 (19.3)	1.09 (0.77–1.55)	1.23 (0.85–1.78)
>0.6–1.2	96 (21.6)	169 (19.3)	1.08 (0.76–1.55)	1.37 (0.94–2.01)
>1.2–2	80 (18.0)	172 (19.6)	0.87 (0.60–1.26)	1.21 (0.80–1.81)
>2–17.1	66 (14.9)	169 (19.3)	0.72 (0.48–1.06)	1.13 (0.74–1.74)
*P* value for trend^c^			0.021/0.042	0.92/0.70
Regular binge drinking (>4 drinks/occasion)				
At age 20 (*n* = 1.320)	130 (29.3)	240 (27.4)	1.14 (0.86–1.52)	1.45 (1.07–1.96)
At age 40 (*n* = 1.305)	118 (26.9)	258 (29.8)	0.85 (0.64–1.13)	1.11 (0.82–1.49)
At age 60 (*n* = 888)	53 (18.2)	122 (20.4)	0.84 (0.59–1.22)	1.03 (0.70–1.51)

Abbreviations: OR = odds ratio, CI = confidence interval.

a Coffee consumption information was missing for one control, and was thus excluded in analyses including coffee consumption.

b The adjusted model includes smoking and/or coffee consumption (in quartiles).

c The first *P* value for trend was based on analyses with the exposure as a continuous variable including the persons from the reference category. The second *P* value for trend was based on analysis whereby the persons from the reference category are excluded.

No association with total and average alcohol consumption and PD risk was found (table 3). Binge drinking was not associated with PD risk with a possible exception of binge drinking at age 20 for which a significantly elevated OR was found after adjusting for smoking (table 3).

### Excess odds ratio models

An excess odds ratio model that included a spline function for total smoking and a linear function for time-since-cessation provided the best fit to our data (AIC: 886.2 compared to an AIC of 900.0 for a model in which the modifying effect of time-since-cessation and duration (or intensity) was set to zero). Including a spline function for time-since-cessation did not further improve model fit (AIC: 888.0). Similar, including an additional modifying function for intensity or duration of smoking did not improve model fit (AIC: 888.2 and 885.5, respectively).

**Figure 1:**
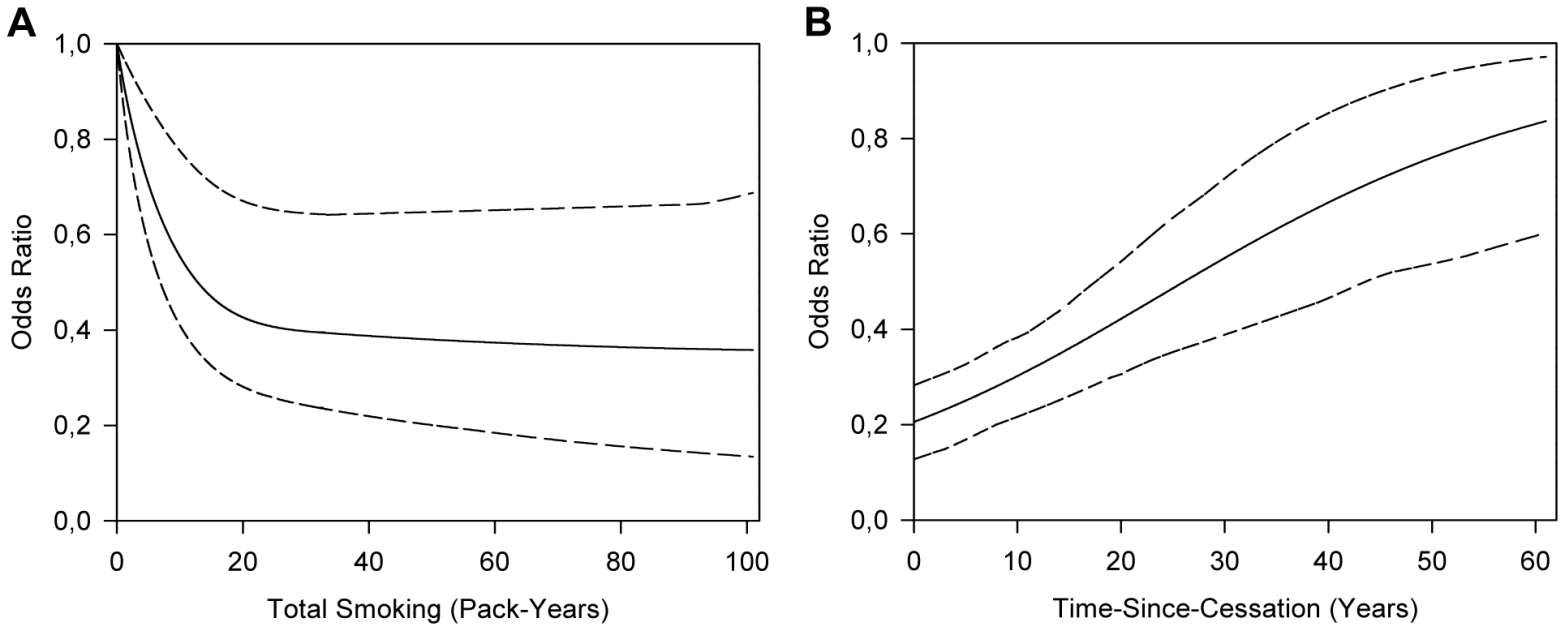
The modifying effects of time-since-cessation on the relation between smoking and PD. The fitted excess OR model with a spline function for total smoking and effect modification by time-since-cessation. 95% confidence intervals were estimated via 100 bootstrap replications. **A**: The OR for different levels of total pack-years, plotted for 21 years-since-cessation. **B**: The OR for different levels of time-since-cessation, plotted for 15 pack-years of total smoking.

In Figure 1 we show the marginal effect of total smoking and time-since-cessation on PD risk from our ‘best’ model, maintaining the other factor in the model at the median level. The plot in figure 1A indicates that the risk of PD decreases with increasing total smoking. Figure 1B indicates that the effect of total smoking on the risk of PD decreases with increasing time-since-cessation.

**Figure 2:**
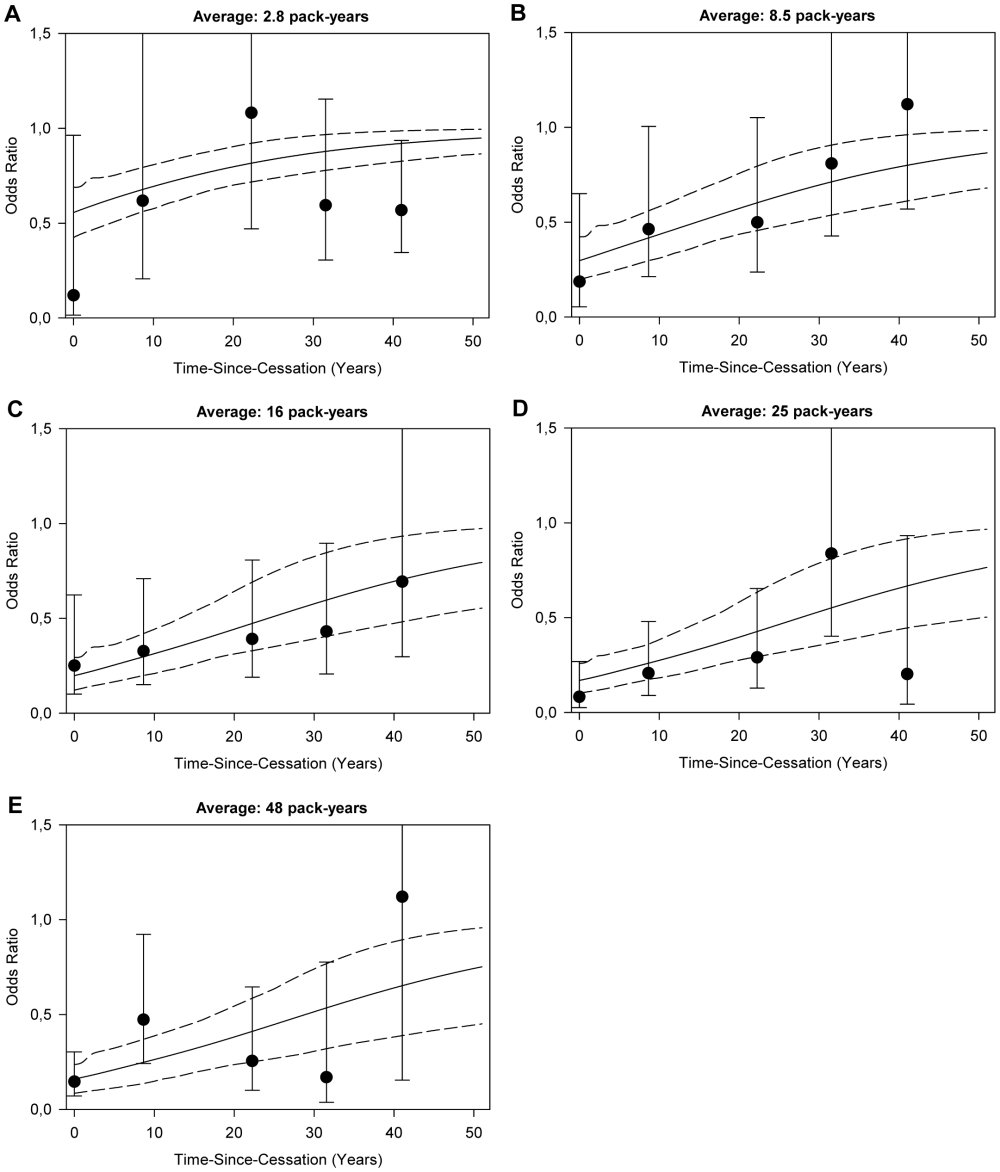
Odds ratios and 95% confidence intervals for time-since-cessation within categories of total pack-years of smoking. A–E: Ever smokers were divided according to quintiles of total-smoking and the effect of time-since-cessation within those groups was plotted. ORs were located at the quintile-specific average time-since-cessation and based on unconditional logistic regression relative to never-smokers adjusted for age, sex and center. The lines represent the predicted ORs and 95% confidence intervals for different levels of time-since-cessation based on the excess OR model plotted for the quintile-specific average pack-years of total smoking.

Ancillary analyses using standard unconditional logistic regression analyses adjusted for age, sex and center corroborate our findings for time-since-cessation from the excess odds ratio model. ORs for time-since-cessation plotted within categories of total smoking follow a pattern similar to the prediction of our excess odds ratio model with ORs being lowest within categories of higher total smoking and shorter time-since-cessation (Figure 2).

## Discussion

In our hospital based case-control study, we found a strong inverse association between cigarette smoking and Parkinson disease, which is in correspondence with earlier studies. We also observed some indication of a potential inverse relation between total and average coffee consumption with PD, but no association with alcohol consumption was found.

Applying a novel modeling method in which the effects of modifying factors are conditioned on total exposure, we found that time-since-cessation has a high impact on the association between total smoking and PD risk. We did not observe effect modification of the effect of total smoking and PD risk by intensity or duration of smoking, indicating that smoking intensity and duration have a similar contribution to reduction of PD risk. The later finding is different from previous findings that smoking duration would be more important than intensity [5], [6].

The applied model in this study is different from previously published models (6,7) in that it models the inverse OR, rather than the OR itself. This modification accommodates the assessment of a protective effect of total pack-years of smoking on PD by avoiding restriction of the parameter estimation range of function f(PCS) (OR>0). We checked the appropriateness of our model specification by *i*) Inversion of case-control status in the dataset and consequential adjustment of the likelihood function to be able to use a standard excess OR model and *ii*) by fitting a standard excess OR model that included a sufficiently flexible function for f(PCS) to accurately assess the asymptotic protective effect of pack-years of smoking [OR = 1+f(PCS)×exp (g(ICS)+h (TSC))]. Both analyses yielded essentially similar results as our preferred less constrained inverse OR model.

Biological mechanisms through which PD is linked to smoking include the nicotinic acetylcholine receptors, which trigger downstream signaling molecules, possibly resulting in protection or delay of the development and progression of PD via effects such as decreased apoptosis, enhanced neuronal survival or modified immune responsiveness [11], [12]. Other possible non-receptor mediated mechanisms of nicotine include modulation of mitochondrial complex I activity or through its action as an antioxidant [12].

As in any case-control study, we should keep in mind that our results might also be (partly) explained by reverse causality. PD patients have been described as having rigid, introverted and low-tempered, and less novelty-seeking personality traits which may lead to lower (or shorter) cigarette or alcohol consumption [13]. By extension, low dopamine levels in individuals long before onset of PD, leading to premorbid Parkinsonian personality traits [14], might have a similar effect. However, many of the studies investigating premorbid personality traits have methodological deficiencies, which make interpretation difficult [15]. Of note, imaging studies have shown nigrostriatal dopaminergic neuronal loss starting to increase less than 10 years before onset of clinical symptoms, suggesting a limited impact on cigarette and alcohol consumption behavior earlier in life [16].

The inverse relationship between smoking and PD could also be the result of a genetic factor related to both smoking behavior and the chance of developing PD, but this hypothesis was not supported by twin studies [17], [18].

Our finding of a relationship between PD risk and time-since-cessation provides some support for a true causal effect, as in the case of reverse causality no direct association with time-since-cessation would be expected. Some of the effect observed for time-since-cessation might in fact be due to smoking duration, as most participants in our study started smoking at a similar age (around 18) and consequently smoking duration and time-since-cessation are inversely correlated (Pearson correlation coefficient: −0.75). However, in a model including modifying factors for both smoking duration and time-since-cessation a strong effect for time-since-cessation, and not for duration, was observed, indicating the observed effect is most likely due to time-since-cessation. In addition, we did not observe a clear increase in the number of cases quitting smoking as a consequence of disease onset in the years before diagnosis (results not shown). As such, results from our analyses of smoking behavior in the aggregate are supportive of a causal protective effect of smoking that diminishes after quitting smoking.

We also observed an inverse exposure dependent association with total coffee consumptions and the average number of consumptions per day and the risk of PD. These associations were however not statistically significant in models adjusted for smoking or in analyses limited to never smokers. Coffee has been shown to be inversely related to PD risk in three meta-analyses [2], [19], [20] and can be explained biologically by the idea that caffeine in coffee is neuroprotective [21]. However, given the relatively large impact of the adjustment by smoking on the observed ORs we cannot rule out that the observed effect is due to residual confounding by smoking.

Contrary to what has been suggested in the literature [22], we did not observe an inverse association with alcohol consumption in our study. We did observe a positive association with PD for regular binge drinking at age 20, suggesting that drinking high amounts of alcohol at a young age may increase the risk for PD. Given that this could be a chance finding this result should be interpreted with caution and needs replication.

A limitation of our study is the low participation rate. The participation was lower among women (for cases 40%) than men (49%), and depended on age. Under the age of 70, the participation of cases was 66%. Sensitivity analysis restricted to persons under age 70 showed similar results as when including all participants (data not shown). About 50% of the non-participants provided a reason for their decline. Health related reasons were reported most frequently, but compared to cases, more controls reported to be not interested.

Another possible limitation of our study is that controls were selected from the same neurology departments as the cases and that the underlying disease mechanism for these non-neurodegenerative conditions may share some characteristics with PD. Repeating the analyses leaving out one control group at a time (based on DBC codes), resulted in almost identical results, suggesting that the results are not driven by one specific control group adding to the validity of our results.

In conclusion, in a case-control study of 444 recently diagnosed PD patients and 876 controls, we found an inverse association of cigarette smoking and coffee consumption but not of alcohol consumption with the risk of PD. In the association with smoking, total smoking and time-since-smoking cessation appear to drive PD risk. No effect modification of total smoking and PD risk by either the intensity or the duration of smoking was observed, indicating that both aspects have an equal contribution to the reduction of PD risk.

These results provide some further insights into the etiology of PD and may support the usefulness of randomized controlled trials investigating the possibility that administering nicotine or nicotine-mimicking drugs to PD patients might be effective in delaying disease progression.
